# Livelihood challenges of single female household heads in the Rohingya and host communities in Cox’s Bazar, Bangladesh during the COVID-19 pandemic

**DOI:** 10.1186/s12889-023-16964-2

**Published:** 2023-10-24

**Authors:** Sameen Nasar, A. S. M. Nadim, Saifa Raz, Abdul Jabbar, Muhammad Riaz Hossain, Bachera Aktar, M Shafiqur Rahman, Sabina Faiz Rashid

**Affiliations:** 1 BRAC James P Grant School of Public Health, BRAC University, 6th Floor Medona Tower, Mohakhali, Dhaka, 1212 Bangladesh; 2 Save the Children, Cox’s Bazar, Bangladesh; 3 Institute of Statistical Research and Training, University of Dhaka, Dhaka, Bangladesh; 4 Liverpool School of Tropical Medicine, Liverpool, UK

**Keywords:** Humanitarian crises, Humanitarian health, Single female household heads, Livelihood, Mental health, Bangladesh

## Abstract

**Background:**

Following the mass influx of Rohingya refugees into Cox’s Bazaar, Bangladesh in 2017, makeshift settlement camps in Ukhiya and Teknaf have been overburdened, leading to livelihood challenges for both Rohingya and host communities. The humanitarian crisis has had adverse effects on vulnerable populations, which include older people, persons with disabilities, adolescents, and single female household heads. Using a subset of a larger dataset on households with most vulnerable groups in both communities, we analysed the effect of the pandemic and lockdown on the livelihood of single female household (HH) heads.

**Methods:**

A cross-sectional household roster survey was designed to collect data from households with most vulnerable groups (MVGs) of host and Rohingya communities from December 2020 to March 2021; 11 host community villages and 10 Rohingya camps purposively selected as per the affiliated intervention of the project. The paper analysed quantitative and qualitative data from the sub-group of single female household heads without any income/low income. Participants were surveyed for their socio-demographic characteristics, COVID-19 experiences and knowledge, food security situation, social experiences and mental health using PHQ-2 test for depression.

**Results:**

We surveyed 432 single female HH heads. Support during the pandemic was reported to be low, with less than 50% of HHs reporting relief meeting their needs; only 36% and 15% of these HHs received rations in camps and host communities respectively. Loan facilities were mostly unavailable and there were reported insufficiencies in food consumption. Over 50% of respondents tested positive on the PHQ-2, a scale used to screen for depression. Further analyses indicates that having a chronic health issue (OR 2.2, 95% CI 1.33–3.66) was positively associated with the PHQ-2 score for Rohingya single females. For host single females, having an ill member in the HH (OR 1.46, 95% CI 1.02–2.08) and the inability to save before the pandemic (OR 1.57 95% CI 1.11–2.23) increased the odds of screening positive for depression.

**Conclusion:**

Our study findings revealed insufficiencies with economic opportunities and food security for single female-headed households, as well as a high rate of positive screening for depression amongst this population. These findings call for a more in-depth understanding of the needs of this group.

**Supplementary Information:**

The online version contains supplementary material available at 10.1186/s12889-023-16964-2.

## Background

The Rohingyas represent the largest group of Muslims in Myanmar. Since 1982, successive governments in Myanmar have refused to recognize them as citizens of the state. The displaced Rohingya population have been accommodated into makeshift settlement camps in Ukhiya and Teknaf, the two sub-districts (Upazila) of Cox’s Bazar in Bangladesh, where there has been periodic movement since 1991, with the largest influx taking place in August of 2017 [1]. Currently, it is officially reported that there are 884,641 Rohingya people, with 48% men and boys and 52% women and girls in Rohingya camps approximately [2]. Prior to the large influx of Rohingyas in 2017, there has been a long history of the host community and Rohingya, where influxes have started since the late 70s [3]. The Cox’s Bazar district, a coastal region, is subject to serious high winds and storm surges leading to floods [4], affecting the livelihoods of the local populations. Despite these influxes providing some economic opportunities for the host community locals [5], the region remains one of the poorest in the country [6]. The Rohingya population is almost twice as large as the host populations; where Ukhiya & Teknaf have a combined population of 456,732 [7], with approximately equal gender distributions (Males = 51%; Females = 49%).

The pandemic and the subsequent lockdown have further escalated challenges for this community and the host communities, following the first case in the camps, which was detected on the 14th of May 2020 [8]. COVID-19 testing results from the first week of January 2021 revealed, out of 25,281 tests, there were 373 cases and 10 deaths; with 50% male and female, with the majority of deaths among children between 0 and 10 years (36%) [9]. Since then, cases have risen, with peaks occurring in February and May, and as of mid-June, there are 1,573 confirmed cases amongst Rohingya refugees [10]. A rapid gender analysis by the Inter Sector Coordination Group (ISCG) reported that pre-existing gender inequalities could exacerbate livelihood challenges in both Rohingya and host communities, where host female headed households without males are less likely to have income generating opportunities [11]. Another report by the ISCG revealed that 34% women and 43% Rohingya men and their families had completely lost their income due to the containment measures for COVID-19 [11]. However, despite basic food and material needs being covered by NGOs, a study by the X-Border Local Research Network found that the average household expenses for the Rohingya population are estimated to be around BDT 7,987, close to three times the average reported income of BDT 2,648 [12]. Such expenses include food items (meat, fish, fruit), religious education and private tutoring for children (BDT 40–600 per month), mobile data (BDT 350–700 per month), medical treatment outside of camps (BDT 2,975 or higher per month), with other expenses on dowry family purchases such as clothes [12]. Therefore, understanding day-to-day livelihood is critical to addressing their needs.

With some evidence of attitudes of women’s role in income generation shifting due to the economic situation in the camps [13], conservative cultural norms still persist and create problems within the household [12], where women negatively describe this greater mobility, stating they have to go out and talk to men, which is not a good thing [13]. In the host community, working women are mostly engaged in the informal economy, where there is little to no social or employee protections, with no financial support during the pandemic [14].

Women, as well as children and trans-genders, face increased barriers accessing health services [11]; as a result, they are more vulnerable to infectious diseases. Furthermore, as women are the primary caregivers in this community, their health status directly affects young children. As mentioned, the rapid gender analysis reveals female-headed households without adult males are less likely to have access to income generating opportunities [14]. This adds to their livelihood challenges, where single female household heads of host communities are at risk of eviction [14]; the lack of jobs leads to reduced incomes, leading to an inability to make room/house payments. Women and girls account for just over 50% of the population in the camps [15, 16], where the highest proportions of the population are single parents or caregivers [17]. Additionally, the constraints of not having any male members of working age not only creates difficulties at the household level, but also creates security challenges [11, 14]. This led single females to take on the role of men, adding to their existing concerns with the onset of COVID-19. Given these issues for single female-headed households, to our knowledge, there is minimum research on this vulnerable group in Rohingya camps and host communities of Ukhiya and Teknaf. In this paper, we aim to present their livelihood challenges, which include economic, food security, social and mental health aspects. Findings of the research will be helpful for policymakers for taking special urgent measures for improving their livelihood.

## Methods

### Study participants & design

Data for this paper (Table 1) is from a larger exploratory mixed-methods study combining both qualitative and quantitative methods to collect data from the most vulnerable groups (MVGs) of host and Rohingya communities; a total of 11 host community villages and 10 Rohingya camps purposively, as the study areas were the project implementation partner’s (BRAC Centre for Peace and Justice) areas where they piloted their interventions [18]. A total of 2,057 households (Host: 1,029, Rohingya: 1,027) were interviewed excluding the non-responses. Through qualitative methods, the aim was to uncover a deeper understanding of experiences of respondents. This paper focuses on the sub-group of single (widow/divorced/abandoned by spouse) female-headed household with\ no income or low income (less than BDT 1,000 per month). Their data was extracted from the survey to understand the livelihood challenges faced by single female-headed household due to COVID-19, which include socio-economic status, food security and impacts on mental health.

**Table 1:** Selected single female headed HHs in Rohingya camps & host communities

	Rohingya		Host		
	N	%		N	%
**Residence**			**Ward**		
Camp 1	80	26.94	Rajapalong Ward 2	64	45.07
Camp 2	64	21.55	Rajapalong Ward 5	25	17.61
Camp 3	29	9.76	Rajapalong Ward 6	27	19.01
Camp 4	35	11.78	Rajapalong Ward 9	26	18.31
Camp 5	27	9.09	**Total**	142	100.00
Camp 6	9	3.03			
Camp 7	14	4.71			
Camp 8	10	3.37			
Camp 9	12	4.04			
Camp 10	17	5.72			
**Total**	297	100.00			

### Data collection

Respondents in the qualitative phase were contacted and identified using purposive, convenient and opportunistic sampling techniques based on pre-set criteria agreed-upon by the larger research team. A total of 4 IDIs with single female household heads were conducted in each community respectively.

### Variables and associated measures

From the collected data, we analysed socio-demographic variables of age, marital status, COVID-19 symptoms, economic status, illness within households, as well as facilities and food security related variables. Administering the PHQ-2, with an additional five items from the PHQ-9, assessed symptoms of depression; following consultation with experts and community leaders, two items were omitted due to the nature of the statement items. The PHQ-2 include items rated on a 4‒point Likert scale ranging from 0 (“not at all”) to 3 (“nearly every day”). The same scale was used for the other five items. Categorical outcomes for PHQ-2 included ‘positive’ and ‘negative’ for depressive symptoms with a total range from 0 to 6, with a cut-off score of 3 or above screening positive for depressive symptoms [19]. This measure screens for possible depression within the last 2 weeks (15 days) prior to the day of data collection. For this study, the PHQ-2 has shown good reliability for exploratory purposes, with an alpha coefficient of 0.74, and an alpha coefficient of 0.76 for all seven items used (See Additional File 1).

### Statistical analysis

All collected data were managed and analysed using STATA versions 13 and 16. Descriptive statistics for single female household heads were computed using frequencies and percentages for all categorical data. Further, bivariate analysis with chi-square test was performed to examine the association of social-economic impacts on single female household head in comparison with the counterpart (male headed household). Internal consistencies of the PHQ-9 items were tested using Cronbach’s Alpha; for PHQ-2, an alpha of 0.74 was obtained, and is considered suitable for exploratory research [20]. Furthermore, these respondents were subject to bivariate analysis to understand possible risk factors associated with a positive screening.

### Qualitative analysis

For qualitative data, after data were checked and crosschecked to confirm validity, the researchers familiarized themselves with the data, coded the transcripts inductively and identified key themes and sub-themes. The coded data were manually consolidated into data display matrices in Microsoft Excel according to key themes and sub-themes, where quotes were extracted and analysed.

### Ethical consideration

The study protocol and all instruments were reviewed and approved by the institutional research review board (IRB- 6 November’20–057) at the BRAC James P Grant School of Public Health, BRAC University. Each respondent was clearly informed that their participation in this study would not affect in them receiving any services and their and their identification details would not be disclosed or published anywhere. Only those who agreed and provided consent to participate in the research were interviewed.

## Results

### Participants

A total of 508 single female-headed households (HH) from both the Rohingya and host communities were taken from the larger household survey (n = 2,057). The table below provides a breakdown of the characteristics of female HH heads in both communities.

The mean age of single female household heads was 40.32 and 44.58 years for Rohingya and host communities respectively (Table 2). In both communities, the majority of the single females had been widowed (77.81%; 59.86%), with a small proportion that remained unmarried (2.74%; 3.52%); there were 14 Rohingya respondents who were still married at the time of survey, and were either in the process of divorce, or their husbands were seeking refuge in another country (Table 2). The majority of HHs in Rohingya camps had 1–2 (46.99%) members, with 3 and 4 members HHs making up 35.52% of HHs respectively. Whereas in the host community, the majority of single female headed households consisted of 3 to 4 members (45.07%) (Table 2). The survey consisted of questions relating to impact as a result of the pandemic and subsequent lockdown; areas include economic impact, food security, water and sanitation, general health and health seeking.

**Table 2:** Characteristics of surveyed single female HH heads in Rohingya camps & host communities

	Rohingya		Host	
	N	%	N	%
**Age (Mean & SD) | CI**	**40.32(14.13)**	**38.86–41.77**	**44.58(14.78)**	**44.13–47.04**
15 to 19	45.75	45.75	48	33.79
20 to 40	26.04	26.04	41	28.86
41 to 50	20.01	20.01	34	23.93
60+	7.67	7.67	18	12.66
**Marital Status**				
Married	14	3.84	28	19.72
Unmarried	10	2.74	5	3.52
Widow/Widower	284	77.81	85	59.86
Divorced	52	14.25	23	16.2
Separated	5	1.37	1	0.7
**HH Composition (Mean & SD) | CI**	**3.02(1.74)**	**2.84–3.20**	**3.43(1.64)**	**3.16–3.70**
1–2 Member HH	172	46.99	44	30.99
3–4 Member HH	130	35.52	64	45.07
5 or more Member HH	64	17.49	34	23.94

### Economic conditions & food security

Qualitative interviews in the formative phase of the project revealed noteworthy impacts on food and economic conditions of Rohingya in the camps. Therefore, the survey focused on key questions around income and food conditions.

Approximately 36% of Rohingya single female headed households (SFHHH) reported receiving rations, almost two times lower than the ‘other’ households received rations (64.31%). Only 15% of host single female-headed households reported receive any sort of ration, which in stark contrast to 85% of other households. About two thirds (75%) and close to half (44%) of SFHHHs in Rohingya and host reported not having regular jobs (Work is usually task based, either for an NGO or a shop owner). This may explain why approximately 38% of the respondents reported a complete stop in income, with 29% reporting a slight decrease. This was much lower for host SFHHHs (8%), which may indicate more difficulties in securing employment for them. Furthermore, when asked for an estimate of minimum monthly income required for HH expenditures, the average was BDT 6,666.2 and BDT 12,926.1 per month for Rohingya and host SFHHHs respectively, with 23.1% of Rohingya and 7.0% of host SFHHHs reporting they could not save any money following expenditure during the outbreak and lockdown periods. This may explain why close half of Rohingya SFHHH respondent’s perception of current HH income is ‘not enough’ (37%) and for other host households it was above 80%. Furthermore, only 21% and 35% of Rohingya and host respondents reported being able to save money before the pandemic (Table 3), which was reported to last 22 and 27 days on average. However, when asked about loans separately, about 36% Rohingya SFHHH respondents reported to take loans, whereas only 14% of host SFHHH were able to source loans. The most common sources of loans for Rohingya SFHHHs were from shops (63%), money-lenders (43%) and NGOs (38%). For host SFHHH the main sources were neighbours (18%), relatives (14%) and shops (12%). Less than 50% of respondents in both communities reported relief meeting their needs (40%; 21%); when asked what was required, majority from both communities reported money and food (Additional File 2).

**Table 3:** Impact of COVID-19 on income & food consumption on single female headed HHs

	Rohingya		Host	
	Single Female Headed HHs	Other HHs	Single Female Headed HHs	Other HHs
	%	%	%	%
**HH Receives Ration**				
Yes	35.61	64.31	15.27	84.73
No	37.5	62.5	13.63	86.37
***p value***	0.025		0.613	
**HH Income Compared to “before pandemic”**				
Same	39.34	60.66	11.81	88.19
Decreased somewhat	27.10	72.90	12.50	87.50
Completely stopped	37.70	62.30	26.96	73.04
Increased	29.17	70.83	7.94	92.06
***p value***	0.006		0.000	
**Ability to save any money after HH monthly expenditure during pandemic**				
Yes	23.08	76.92	6.90	93.10
No	35.92	64.08	14.33	85.67
***p value***	0.177		0.112	
**Perception of Monthly HH Income during pandemic**				
More than enough	40.00	60.00	14.08	85.92
Enough/adequate	27.95	72.05	2.58	97.42
Not enough	37.03	62.97	17.31	82.69
***p value***	0.084		0.000	
**Availed Loans**	35.8%	64.2%	14.1%	85.9%
***p value***	0.94		0.78	
Source of loans during pandemic*				
Neighbours	38.7	61.30	17.72	86.14
Relatives	33.14	66.86	13.86	86.14
Shop	62.50	37.50	11.54	88.46
Friends	22.86	77.14	5.80	94.20
Money Lender	42.86	57.14	8.33	91.67
Other NGO	37.50	62.50	0.00	0.00
***p*** **value**	0.057		0.315	
**Effect on food consumption compared to “before pandemic”**				
It has stayed about same	37.07	62.93	7.93	92.07
It has increased	21.74	78.26	15.22	84.78
It has declined	34.89	65.11	15.51	84.49
***p*** **value**	0.287		0.014	
If declined, now consuming less of*				
Meat, poultry & fish	34.77	65.23	15.96	84.04
Fruits	37.21	52.79	14.29	85.71
Dark green leafy vegetables	36.09	63.95	19.35	80.65
Milk/Dairy	32.05	67.95	15.73	84.27
Other vegetables	39.78	60.22	20.00	80.00
Eggs	27.55	72.45	17.87	82.13
Fast food/Outdoor food	27.91	72.09	17.65	82.35
Nuts & seeds	33.33	66.67	9.09	90.91
Grains, white roots, tubers & plantains	30.85	69.15	16.13	83.87
Pulses (Beans, peas & lentils)	25.00	75.00	14.29	85.71
***p*** **value**	0.143		0.208	

p values were generated using **χ**^**2**^ **Tests**

Income effects of the pandemic affected the nutritional status of HHs differently, with less than half of Rohingya SFHHHs reporting food consumption staying the same (37.1%) as it did before the pandemic, and 35% reporting a decline. Also, close to two-thirds of ‘other’ Rohingya households reported a decline, though this was statistically non-significant, however, this may indicate insufficiency in aid/relief distribution. Around 15% Host SFHHHs reported a decline compared to 85% of other households, which may indicate an overall reduction in food supplies within host communities. Vegetables, nuts and grains, meats and fruits were the most reported food items for decline amongst Rohingya SFHHHs. For other HHs it was mainly fast food & eggs, grains, nuts, dairy and meats. Similar trends for consumption decline were reported in the host community. These shortages or reductions in consumption may have resulted in poorer health outcomes, leading to, or adding stress to an already difficult situation with regards to individual and HH livelihood.

### Depression screening and symptoms

Mental health status may be exacerbated in the wake of livelihood challenges or may be a result of such challenges. Given the vulnerable groups in our research, it is possible that mental health disorders are prevalent, and made worse with pandemic and lockdown. Using a truncated PHQ-9 scale, we looked for depressive symptoms, and utilized the PHQ-2 as primary screener for depression. Overall, over half of the single females HH heads screened positive (Table 4).

**Table 4:** Depression screening and PHQ details

	Rohingya Communities				Host Communities				
	Single Female HH Heads		Others HH Heads		Single Female HH Heads		Others HH Heads		
	N	%	N	%	N	%	N	%	
**Depression screening using PHQ-2**									
Positive ( > = 3)	194	53.59	478	72.87	103	73.05	681	77.83	
Negative (3<)	168	46.41	178	27.13	38	26.95	194	22.17	
**Featured PHQ-9 Items**									
**PHQ1**: Interest/pleasure									**PHQ2**
Not at all	80	22.79	96	29.63	14	12.07	96	29.63	
Several days	132	37.61	111	34.26	38	32.76	111	34.26	
More than half of the days	94	26.78	66	20.37	36	31.03	66	20.37	
Nearly everyday	45	12.82	51	15.74	28	24.14	51	15.74	
**PHQ2**: Feeling down/depressed/hopeless									
Not at all	52	14.81	56	17.28	9	7.76	56	17.28	
Several days	135	38.46	134	41.36	41	35.34	134	41.36	
More than half of days	112	31.91	84	25.93	41	35.34	84	25.93	
Nearly everyday	52	14.81	50	15.43	25	21.55	50	15.43	
**PHQ3**: Trouble **s**leeping									
Not at all	81	23.08	99	30.56	15	12.93	99	30.56	
Several days	157	44.73	121	37.35	37	31.9	121	37.35	
More than half of the days	73	20.8	67	20.68	30	25.86	67	20.68	
Nearly everyday	40	11.4	37	11.42	34	29.31	37	11.42	
**PHQ5**: Poor appetite/overeating		b							
Not at all	129	36.75	151	46.60	19	16.38	151	46.60	
Several days	143	40.74	100	30.86	48	41.38	100	30.86	
More than half of the days	55	15.67	53	16.36	34	29.31	53	16.36	
Nearly everyday	24	6.84	20	6.17	15	12.93	20	6.17	
**PHQ6**: Feeling Bad/Failure									
Not at all	179	51.0	163	50.31	46	39.66	163	50.31	
Several days	72	20.51	71	21.91	32	27.59	71	21.91	
More than half of the days	44	12.54	42	12.96	18	15.52	42	12.96	
Nearly everyday	56	15.95	48	14.81	20	17.24	48	14.81	
**PHQ7**: Concentrating									
Not at all	210	59.83	165	50.93	61	52.59	165	50.93	
Several days	92	26.21	103	31.79	32	27.59	103	31.79	
More than half of the days	39	11.11	40	12.35	17	14.66	40	12.35	
Nearly everyday	10	2.85	16	4.94	6	5.17	16	4.94	
**PHQ8**: Moving/Speaking slowly									
Not at all	250	71.23	224	69.14	66	56.9	224	69.14	
Several days	56	15.95	60	18.52	23	19.83	60	18.52	
More than half of the days	20	5.7	20	6.17	15	12.93	20	6.17	
Nearly everyday	25	7.12	20	6.17	12	10.34	20	6.17	

Overall, over half of the Rohingya SFHHHs respondents tested positive (54%) on the PHQ-2. About 73% of host single female HH heads were positive. Responses across items were mixed, however, notable findings include over a third of respondents (household heads) across households in both communities reported losing interest or pleasure in doing things over ‘several days’, also single female HH heads in both communities reported feeling down or depressed over ‘several days’ or ‘More than half of the days’ was at a high frequency. For other PHQ-9 items, high responses for single female HH heads were recorded for ‘trouble sleeping’ and ‘poor appetite’ problems over ‘several days’ or ‘more than half of the days’; adding to issues regarding food security and HH finances. The highest reported symptom felt ‘nearly everyday’ amongst Rohingya SFHHHs were symptoms of ‘feeling down, depressed or hopeless’ (15%) and ‘Feeling bad about yourself—or that you are a failure or have let yourself or your family down’ (16%) (Table 4). For host SFHHHs the highest reported symptom felt ‘nearly everyday’ were ‘losing interest/pleasure’ (24%), ‘Feeling down/depressed/hopeless’ (22%), ‘Trouble falling or staying asleep, or sleeping too much’ (29%), and ‘Feeling bad about yourself—or that you are a failure or have let yourself or your family down’ (17.2%) (Table 4).

### Socio-demographic correlates of depression screening

Using the PHQ-2 depression screener, bivariate analysis was conducted with selected socio-demographic variables to get a better understanding of the contributors towards mental health of respondents, therefore, a rough proxy of their overall livelihood.

According to the bivariate analysis, having some sort of chronic health issue increased the odds of testing positive on the PHQ-2 for all HH heads except ‘other’ heads in the host community; amongst single Rohingya female HH heads odds were twice as higher compared to those not having chronic illness and was statistically significant (OR 2.2, 95% CI 1.33–3.66) (Table 5). Increasing age had no significant effects on testing positive for HH heads across all communities. Access to safe bathing facilities increased odds of testing positive for all HH heads, however these odds were not statistically significant. With regards to COVID-19 symptoms and illnesses in the HH, experiencing symptoms on HH members increased odds for HH heads in the Rohingya camps, which were non-significant. However, having an ill member in the last 2 weeks (15 days of the survey) significantly increased the odds for other HH heads in the host community (OR 1.46, 95% CI 1.02–2.08); single Rohingya HH heads had the highest odds overall, this was not statistically significant. Less than 50% of HH heads in Rohingya camp reported social activities to be affected by the pandemic; reporting an effect on social activities significantly increased the odds for ‘other’ Rohingya HH heads of testing positive (OR 1.57, 95% CI 1.11–2.23) (Table 5). Though over 90% of Rohingya HH heads reported not being able to save before the pandemic, this did not increase the odds positive PHQ2 testing. However, for HH heads in the host community, it significantly increased the odds of testing positive, with single female HH heads having savings twice the odds (OR 4.05, 95% CI 1.72–9.51) of ‘other’ HH heads (OR 1.81, 95% CI 1.31–2.50) to test positive (Table 5). Approximately 56% single Rohingya female HH heads reported not receiving relief, 9% points higher than other HH heads, though this did not have significant effects on positive PHQ2 screening. This was similar for host HH heads; despite over two-thirds of host HHs reported not receiving relief. There were similar findings regarding consumption and food shortages, though HHs across both communities reported declines, and over 50% reported running out of food due to insufficient resources.

**Table 5:** Socio-demographic, economic & food security correlates of depression screening

	Rohingya Communities								Host Communities								
	Single Female HH Heads				Other HH Heads				Single Female HH Heads				Other HH Heads				
	%	*p*-value	Unadjusted OR	95% CI	%	*p*-value	Unadjusted OR	95% CI	%	*p*-value	Unadjusted OR	95% CI	%	*p*-value	Unadjusted OR	95% CI	
**Age**																	
15 to 20 (Ref)	5.82				9.48				1.42				10.97				
21 to 35	40.17	0.45	0.7	0.28–1.76	45.11	0.31	0.69	0.34–1.40	32.62	0.76	1.25	0.31–5.08	53.94	0.04	0.49	0.24–0.97	
36 to 50	26.32	0.53	0.73	0.28– 1.91	15.44	0.04	0.44	0.21–0.96	29.08	0.42	1.82	0.43–7.69	19.54	0.00	0.27	0.13–0.55	
51 to 64	24.65	0.84	1.10	0.42–2.89	12.39	0.08	0.48	0.22–1.08	29.79	0.12	3.33	0.74–14.98	7.43	0.01	0.30	0.13–0.71	
65 & above	3.05	–	1.0	–	17.58	0.03	0.42	0.20–0.90	7.09	–	1.00	–	8.11	0.00	0.24	0.11–0.55	
**HH Size**																	
1–2 Member HH	46.96		Ref		16.62		Ref		31.21		Ref		6.17		Ref		
3–4 Member HH	35.64	0.20	0.87	0.55–1.38	34.76	0.42	0.96	0.69–1.87	45.39	0.15	0.96	0.41–2.26	39.31	0.15	1.59	0.85–2.98	
>=5 Member HH	17.4	0.26	0.89	0.50–1.59	48.63	0.62	1.17	0.85–2.22	23.4	0.08	1.17	0.42–3.30	54.51	0.08	1.73	0.94–3.20	
**Chronic Health Issues**																	
No	75.96		Ref		82.12		Ref		66.67		Ref		84.67		Ref		
Yes	24.04	0.002	2.21	1.33–3.66	17.88	0.29	1.57	0.69–3.59	33.33	0.91	1.59	0.70–3.64	15.33	0.66	0.91	0.59–1.40	
**Smoke cigarettes or consume betel leaf**																	
No	36.90		Ref		22.42		Ref		42.96		Ref		41.2		Ref		
Yes	63.11	0.111	0.71	0.46–1.08	77.58	0.51	0.87	0.57–1.32	57.04	0.08	1.98	0.93–4.19	58.8	0.58	0.91	0.66–1.26	
**Access to drinking water within premises**																	
Yes	82.04		Ref		73.48		Ref		77.3		Ref		78.35		Ref		
No	17.96	0.44	0.81	0.47–1.38	26.52	0.15	1.34	0.90–2.01	22.7	0.53	0.76	0.32–1.80	21.65	0.69	1.08	0.73–1.60	
**Toilet within premises**																	
Yes	76.50		Ref		73.52				81.69				86.44				
No	23.50	0.06	1.62	0.98–2.66	26.48	0.29	1.24	0.83–1.85	18.31	0.64	1.27	0.47–3.45	13.56	0.19	0.74	0.48–1.16	
**Safe bathing facilities**																	
Yes	84.97		Ref		86.84		Ref		77.14		Ref		77.9		Ref		
No	15.03	0.32	1.34	0.75–2.41	13.16	0.89	1.04	0.62–1.73	22.86	0.45	1.44	0.56–3.67	22.1	0.52	1.14	0.77–1.68	
**Cooking facilities within premises**																	
Yes	97.27		Ref		98.18		Ref		98.58		Ref		98.42		Ref		
No	2.73	0.69	1.30	0.36–4.67	1.82	0.62	0.74	0.22–2.48	1.42	–	1.00	–	1.58	0.23	0.50	0.17–1.52	
**Anyone experience C-19 symptoms (Last 1 month)**																	
No	18.63		Ref		17.45		Ref		16.43		Ref		21.16		Ref		
Yes	81.37	0.23	1.39	0.81–2.38	82.55	0.1	1.51	0.93–2.46	83.57	0.32	0.62	0.24–1.61	78.84	0.32	1.23	0.82–1.84	
**Any HH members ill in last 15 days**																	
No	78.96		Ref		72.62		Ref		66.9		Ref		67.27		Ref		
Yes	21.04	0.08	1.72	0.63–1.72	27.38	0.24	1.27	0.85–1.88	33.1	0.33	0.68	0.31–1.48	32.73	0.04	1.46	1.02–2.08	
** C-19 affect regular social activities**																	
No	59.29		Ref		62.63		Ref		88.73		Ref		83.65		Ref		
Yes	40.71	0.003	0.53	0.35–0.81	37.37	0.01	1.57	1.11–2.23	11.27	0.87	0.90	0.27–2.99	16.35	0.67	1.10	0.72–1.67	
**Able to save money before pandemic**																	
Yes	2.47		Ref		6.35		Ref		21.28		Ref		35.21		Ref		
No	97.53	0.91	0.93	0.24–3.51	93.65	0.03	0.35	0.13–0.89	78.72	0.00	4.05	1.72–9.51	64.79	0.00	1.81	1.31–2.50	
**Receive support (Relief)**																	
Yes	44.45		Ref		62.78		Ref		32.39		Ref		22.94		Ref		
No	55.55	0.66	0.91	0.58–1.41	37.22	0.02	0.65	0.46–0.92	67.61	0.90	0.95	0.43–2.11	77.06	0.60	0.90	0.61–1.32	
**Food consumption during pandemic**																	
Stayed about the same	49.73		Ref		46.75		Ref		12.68		Ref		23.59		Ref		
It has increased	1.37	0.68	1.47	0.24–8.99	2.72	0.05	7.54	0.99–57.66	9.86	0.77	1.25	0.27–5.70	8.8	0.14	0.64	0.35–1.15	
It has declined	48.91	0.22	1.30	0.86–1.96	50.53	0.02	1.51	1.07–2.15	77.46	0.49	1.46	0.50–4.27	67.61	0.92	0.98	0.67–1.44	
**Run out of food due to a lack of resources/money**																	
No	33.15		Ref		35.52		Ref		17.02		Ref		43.43		Ref		
Yes	66.85	0.06	1.52	0.98–2.35	64.48	0.74	1.06	0.74–1.52	82.98	0.79	1.14	0.43–3.02	56.57	0.11	1.30	0.94–1.79	

### Qualitative interviews

#### Rohingya Community

Interviews provided a deeper insight into the problems faced by female household heads. These categories of respondents mostly faced income loss during the pandemic. A twenty-two-year-old widow in the Rohingya camps detailed her struggles of not having a job and taking care of her father.

*“We can’t move outside easily, can’t earn money & eat that we did in Myanmar. People of this country can earn by job but we the Rohingya people cannot… The ration we got from government is not enough for our-self. If we could earn, we can eat meat & fish regularly. So we cannot buy & eat. During covid-19, I have been suffering a lot. If I had a son or a husband, they could bring meat & fish and whatever I wished for… I don’t have any support from my own house because my mother died & my father is old enough.”* (35-year-old Single Female, Rohingya Camps).

This respondent highlights the importance of having a male member in the family, and reiterates the need for additional income, as the rations provided in the camps is not deemed sufficient. Another single female household head in the Rohingya camps echoed similar sentiments.

*“I mean, I had trouble before that I didn’t get rations then…if I want to eat a little good meat, I can’t bring it, even if I want to eat a little big fish, I can’t, even if I want to take clothes, I can’t wear it. So I’m in trouble now”* (48 years old, Rohingya).

According to this respondent, she faced troubles in sources before rations were being distributed. She reported problems with sourcing food, especially protein sources, and also had problems getting clothes. Another respondent also mentioned the rations provided were not enough, and that without money, it would be difficult to get her sisters’ married. She also mentioned to us that there was often a significant trade-off between spending for money and food.

*“In case of disease & treatment, I also feel helpless. It is additional expenditure & costly sometimes. As I do not have any male or earning member in my family, it is almost impossible for me to bear the extra cost. Suppose, last time I had to buy medicines that costs 100 Taka and later I passed few days in a tough situation because I could buy vegetables for those days.”* (20-Year-old Single Female, Rohingya Camps).

This again highlights the importance of male members for these families in these settings, as it is often the case, most jobs available in the camps are not feasible for females; either due to the physical nature of the jobs, or the frequent social interaction required with other men. All the respondents mentioned inadequate food resources, indicating that, rations and relief do not provide enough for families, and may be short in protein rich foods. Furthermore, a woman going outside of the house is usually looked down upon within the Rohingya community; they face harassment as a result.

#### Host community

Single female household heads in the host communities who were already subject to financial problems faced further distress as a result of the pandemic. As one widow details:

*“Before corona, everything was less expensive. We were able to buy things with 5 taka, but now we can’t. Now we can eat only one item. Things are difficult like that. I am not the only one suffering everyone is in the same condition… I went to Cox’s Bazaar last month. Me and my daughter did some tests, it took no less than 10,000 Taka. So, our food expenses went there”* (45-Year-Old Single Female, Host).

This respondent highlights that price hikes were not limited to food, but also medical services, where medical service providers increased prices to capitalize on the situation. Furthermore, she states that the fear of going outside of her local neighbourhood subjects her to the local market, where prices have significantly increased because of the lockdown:

*“We couldn’t go to Ukhiya much of fear. There were shops near the roadside, but everything was 10-fold in prize still we had to buy everything from there. Because we couldn’t go because of the lockdown.”* (45-Year-Old Single Female, Host).

Others reported spending only on the very essential necessities, nothing more.

*“In these troubled time I don’t spend much. If I wanted to eat something, I couldn’t because it was too expensive. If I want to buy something, I couldn’t. Even if I need it, I don’t buy it. I buy what is essential. I have to eat; without necessity, I don’t buy extra.”* (32-Year-Old Single Female, Host).

Another single female household head from the community detailed her financial struggles and subsequently, her mental distress because of it. Where, she was unable to source enough food off savings for her and her child.

*“I drank water to keep myself full. I only had 3,000 taka saved! Sometimes I used to eat less and keep the rest for my children. I have to assure my children that they can eat properly once I start earning again. This makes me upset and gives me a lot of mental stress (40-Year-Old Single Female, Host)*.

This respondent reported the distress caused by not being able to fully provide proper nutrition for her child. Additionally, she reported that once her rations from the local area’s union finished, she had to depend on others.

*I got 2,500 taka, 10kg rice, potato and pulses from the Union Member, and this only lasted one month with these. I went to my neighbour’s house for help as well, for sanitizers or soap. But I was scared because of corona all the time when I people during every day because of it”* (40-Year-Old Single Female, Host).

She also reported a constant fear of COVID-19, which added to her distress. From qualitative interviews it becomes clear that the single female HH heads from both communities face reduced employment opportunities, rations, and increased market prices, therefore am increased risk of food insecurity, where, in the camps, insufficient aid such as rations had increased existing vulnerabilities. In the host communities, economic distress was a major factor influencing their livelihoods. Furthermore, the need for male members in a household is very important; males are easily able to get and work difficult jobs and are also less likely to get harassed.

## Discussion

In this study we present findings of single female household heads in Rohingya camps and the host communities of Ukhiya Cox’s Bazar, from a larger household roster survey. Overall, regarding food security, over half of the respondents reported a decline in consumption, with single and ‘other’ headed HHs in Rohingya camps reporting the greatest decline together with ‘other’ headed HHs in the host community. Almost three quarters of households reported running out of resources to get food at some point, where single female headed HHs in both communities received less rations. Households in Rohingya camps reported an estimated average of BDT (Bangladesh Taka) 6,666 to meet household expenses, close to the figure reported in other studies [12]. For host HHs, it was twice as much, given their expenses with rent, utilities, food, and education. With the estimate for minimum monthly income, and aid not meeting all HH needs, it became evident that other means of income generation were required. Household heads in both communities did not have regular jobs, this compounded financial and food security issues for single female HH heads as they received less rations than other HHs.

The prevalence of possible depressive symptoms is high, as two thirds of Rohingya respondents tested positive, and over 70% of host respondents tested positive on the PHQ-2 scale; approximately 20% more host community single female headed HHs tested positive than their Rohingya counterparts. Furthermore, positive screening was significantly associated with reporting for a chronic health issue and running out of food for Rohingya single female HH heads. A decline in food consumption and reduced social activities were significantly associated with increased odds of positive screening for ‘other’ HH heads in the Rohingya camps. Among host respondents, the inability to save before the pandemic resulted in significant odds of positive PHQ-2 screening, where the odds were double for single female HH heads. Qualitative interviews provided insights regarding insufficiency with relief and lack of job opportunities, and the hardships associated with financial problems.

### Camp stressors and impacts on livelihood

To understand livelihood issues, it is important to acknowledge the stressors on Rohingya population both in Myanmar and Bangladesh, a study by Riley and colleagues measured systematic human rights violations through a 23 item scale, it ranked obtaining citizenship, using the name ‘Rohingya’, public group meetings, religious practices, marriage and securing jobs as some of the most severe violations [21]. The study also details daily stressors in Myanmar and Bangladesh, with income issues reported as the main stressors in both Myanmar (30%) and Bangladesh (95%); food issues (79%), education (72%), physical health (62%) were also highly reported [21]. Also, like the findings in our study, fair access to aid was also reported as a problem (47%) [21], this indicated either insufficiencies or possible exploitation at the camp level. The study also reported high proportion of respondents crossing mental health composite scores for post-traumatic stress disorders (PTSD) (61.2%) and emotional distress (84%); age and sex were significantly associated towards predicting PTSD, where daily stressors in Bangladesh significantly predicted functioning difficulty, as well as anxiety and depression [21]. Increasing age increased the odds of positive PHQ-2 for respondents in the 51 to 64 age brackets, despite non-statistical significance. It is important to note, another study on post-displacement effects on Rohingya individuals in camps found an increase in traumatic stress and age, with a 47% prevalence of severe post-traumatic stress symptoms [22]. The study also found physical and sexual abuse pre-displacement to be significantly associated with an increase in mental health symptoms [22] and has also been described in the qualitative component of another study, where lack of food often leads to abuse from husbands [23]. Also, sufficient aid and paid jobs in camps (adjusted prevalence ratio) reduced the risk of developing mental health symptoms [22]. Another study on the elderly Rohingya population in camps revealed a 41% prevalence of depression symptoms, where living alone increased the odds of developing depression symptoms by four and half times (aOR 4.58, CI 1.58‒12.3) [24]; results in this study showed decreased odds of testing positive for possible depression with increasing number of family members, however, this was not statistically significant.

### Host community struggles and livelihood

Following the increased rates of influx of Rohingya refugees since 2017, there have been significant effects on the host communities, as refugees have settled in and around host communities. As a result, there have been socio-economic challenges, as documented by study by Ullah and colleagues, which reported a 38% decline in the annual income of host community households [25], but for households with any sort of farming as the main profession, annual household income increased by 28% [25]. Furthermore, an analysis of residential satisfaction of host communities revealed highly positive correlations of residential satisfaction were social crime, cleanliness, pollution, and water supply [26]. They also find significant relationships with environmental indicators and residential satisfaction, where, living near camps leads to dissatisfaction, and length of time in residence shows a significant inverse relationship to satisfaction, but for those whose work opportunities have remained the same, satisfaction levels are higher [25]. Therefore, effects of the refugee camps on the host population differ based on socio-economic conditions and different groups of people. Regarding single female headed HHs, it was found that they are less likely have access income generating opportunities [14], where basic needs such as food were predicted to disproportionately affect women, with pregnant and lactating women at greater risk of under–nutrition [14]. Our study found that the greatest impact for single female headed HHs in the host communities was the inability to save before the pandemic, this was an outcome of the poor economic conditions of the region, where inadequate infrastructure, poor roads and limited manufacturing industries contribute to poverty in the district [27]. Qualitative interviews revealed that, a combination of no savings and travel restrictions subjected single female HH heads to local markets which experienced price hikes on goods, therefore, leading them to spend less on food, compounding their existing food insecurity. It was previously found that female-headed households are considerably more affected by food insecurity than male-headed households. Women in this region culturally engage less in income-earning activities resulting in fewer economic opportunities, leading them to be more reliant on more insecure livelihood activities [27].

### Livelihood implications for single female household heads in Cox’s Bazar

Given the persistent health issues of Rohingya refugees in Bangladesh, which include, and are not limited to, non-communicable diseases (NCDs), nutritional deficiencies of women and adolescent girls, gender-based violence and high prevalence of NCD risk factors (smoking, tobacco product consumption) [28], the pandemic and lockdown have exacerbated these existing issues and has created new challenges with regards to delivery and proper allocation of care. Relief distribution inefficiencies during and after the pandemic may have had greater impacts on the livelihoods of single females, adding to existing challenges of economic and food security to a vulnerable group that is not prioritized by programmatic interventions. Pre-displacement stressors are inevitable, together with day-to-day issues, mental health needs are not sufficiently addressed. Furthermore, the longer the time period spent inside of refugee camps, as on-going research from other camps has suggested, will only increase the odds of mental health crises [29] exacerbating the stressors faced by this displaced population [30], especially single female HH heads. Furthermore, the World Food Programme’s (WFP) Refugee influx emergency vulnerability assessment’s (REVA) determinants for host community vulnerability are HHs led by women, absence of a working member and a male bread-winner [31]. In contrast, for the Rohingya community, HHs lead by women was not seen as a determinant of vulnerability [31]. However, our study finds that similar livelihood challenges exist in terms of food supplies, where the financial impact on mental health was greater for host single female HH heads.

To gain a better understanding of livelihood challenges of this group, we recommend further research to explore relief/aid inefficiencies. In addition, mental health disorders such as anxiety, depression, and PTSD for single female HH heads should be looked into at more depth, as there may be underlying conditions which may worsen the disorders for this population, who are already dealing with humanitarian context.

### Recommendations & implications

Our findings indicate that there may be a high prevalence of mental health disorders amongst single female HH heads in the refugee camps and host communities, as well as heads of ‘other’ households in the host community. In addition to further studies to better understand the needs of this vulnerable group, there needs to an increased focus by primary health care centres (PHC) and NGOs operating within and outside of the camps for this group. More in-depth qualitative studies should be conducted, as there is little to no knowledge on cultural and linguistic elements of mental health of Rohingyas [32]. This will assist in communication, making current mental health and psychosocial (MHPSS) interventions more efficient in identifying cases that are in need of urgent attention. For the host community, there needs to be an increased focus on monitoring efforts, prioritizing single female HH heads and their economic opportunities and food security, as well as their mental health.

Given the challenges during the pandemic regarding access and safety measures, using a culturally sensitive (though religious leaders) approach, which may also provide geographic benefits can be useful, as was found in a health seeking and mental health study of Syrian refugees in Lebanon [33]. Furthermore, it is important to acknowledge key protective factors that have been established and provide options to this vulnerable group in terms of safe economic opportunities or sufficient relief.

### Strengths and limitations

The main strength of this study, to our knowledge, is that it looks into households with vulnerable groups as the primary target. This allowed us to collect data from all members of the household, focusing on pre-defined vulnerable groups, providing a preliminary understanding of a group that has had very little attention on them (single female household heads).

The nature of the survey did not allow time for a more focused data collection, as the HH roster was included. Furthermore, the mental health assessment was screener, and a full scale was not used.

## Conclusion

Our study findings revealed insufficiencies with economic opportunities and food security of single female-headed households, as well as a high rate of positive screening for depression amongst this population. There was a significant association with reporting chronic health issues, which may be in the form of NCDS, and are affected by livelihood conditions of this vulnerable group. These findings bear important public health research and programmatic implications in this humanitarian setting and calls for a more in-depth understanding of the needs of this group, as well as the mental health of Rohingya refugees to assist current interventions.

### Electronic supplementary material

Below is the link to the electronic supplementary material.

Supplementary Material 1

Supplementary Material 2

## Data Availability

The datasets used and/or analysed during the current study are available from the corresponding author on reasonable request.

## List of abbreviations

BDT: Bangladesh Taka
CI: Confidence Interval
HH: Household
ISCG: Inter Sector Coordination Group
MHPSS: Mental Health and Psychosocial
MVGs: Most Vulnerable Groups
OR: Odds Ratio
aOR: Adjusted Odds Ratio
PHQ-2: Patient Health Questionnaire-2
PHQ-9: Patient Health Questionnaire-9
PTSD: Post-Traumatic Stress Disorders
PHC: Primary Health Care-centres
REVA: Refugee Influx Emergency Vulnerability Assessment
SFHHH: Single female headed households
NCD: Non-Communicable Diseases
WFP: World Food Programme
