# Supplementary material for: Livelihood challenges of single female household heads in the Rohingya and host communities in Cox’s Bazar, Bangladesh during the COVID-19 pandemic
Source: BMC Public Health. 2023 Oct 24;23:2084. doi: 10.1186/s12889-023-16964-2 (PMC10599043; doi:10.1186/s12889-023-16964-2)
Supplement: Supplementary file 1 — Supplementary Material 1 [file 12889_2023_16964_MOESM1_ESM.pdf]

Table 1: Determining cut-off Scores for PHQ-2

Cutpoint	Sensitivity	Specificity	Correctly Classified	LR+	LR-
(>= 0)	100.00%	0.00%	54.93%	1.0000	
(>= 1)	100.00%	23.44%	65.49%	1.3061	0.0000
(>= 2)	100.00%	47.66%	76.41%	1.9104	0.0000
(>=3)	100.00%	100.00%	100.00%		0.0000
(>=4)	32.05%	100.00%	62.68%		0.6795
(>= 5)	17.31%	100.00%	54.58%		0.8269
(>= 6)	0.00%	100.00%	45.07%		1.0000
(> 6)	100.00%	0.00%	54.93%	1.0000	
