# Supplementary material for: Livelihood challenges of single female household heads in the Rohingya and host communities in Cox’s Bazar, Bangladesh during the COVID-19 pandemic
Source: BMC Public Health. 2023 Oct 24;23:2084. doi: 10.1186/s12889-023-16964-2 (PMC10599043; doi:10.1186/s12889-023-16964-2)
Supplement: Supplementary file 2 — Supplementary Material 2 [file 12889_2023_16964_MOESM2_ESM.pdf]

Table 2: Relief status and requirements of Rohingya and Host Communities

	Rohingya		Host	
	**Single Female Headed HHs**	**Others**	**Single Female Headed HHs**	**Others**
	**%**	**%**	**%**	**%**
**Did relief meet needs of HH**				
Yes	39.54	60.46	21.11	78.89
No	34.56	65.44	16.98	83.02
***p* value**	0.192		0.420	
**Relief required by HH**				
Money	38.66	61.34	14.41	85.59
Food	34.70	65.30	16.84	83.16
Treatment for health issues	29.29	70.71	14.72	85.28
Job/work opportunities	29.10	70.90	14.36	85.64
Toilet facility	28.24	71.76	8.87	91.13
Tube well	31.02	68.98	11.18	88.82
House/Accommodation	44.36	55.64	15.90	84.10
Medicine/Drugs	29.46	70.54	11.54	88.46
Soap/Toiletries	34.33	65.67	15.00	85.00
Household essentials (brushes, brooms, pot & pan)	37.29	62.71	15.00	85.00
No support required	33.33	66.67	0.00	100.00
Diagnostic testing	60.00	40.00	0.00	100.00
Skill developing items (tailoring machine/books)	0.00	100.00	16.67	83.33
Bed cloth/comforter	0.00	100.00	25.00	75.00
Others	6.67	93.33	20.00	80.00
***p* value**	0.263		0.072	
